# Guided Internet-delivered cognitive behavioural therapy in patients with non-cardiac chest pain – a pilot randomized controlled study

**DOI:** 10.1186/s13063-016-1491-1

**Published:** 2016-07-26

**Authors:** Ghassan Mourad, Anna Strömberg, Egil Jonsbu, Mikael Gustafsson, Peter Johansson, Tiny Jaarsma

**Affiliations:** 1 Department of Social and Welfare Studies, Linköping University, Kungsgatan 40, SE-601 74 Norrköping, Sweden; 2 Department of Medical and Health Sciences, Linköping University, Linköping, Sweden; 3 Department of Cardiology, Linköping University Hospital, Linköping, Sweden; 4 Department of Psychiatry, More and Romsdal Hospital Trust, Molde, Norway; 5 Department of Neuroscience, Norwegian University of Science and Technology, Trondheim, Norway

**Keywords:** Cardiac anxiety, Cognitive behavioural therapy, Fear of body sensations, Internet-delivered, Non-cardiac chest pain, Randomized controlled study

## Abstract

**Background:**

Patients with recurrent episodes of non-cardiac chest pain may experience cardiac anxiety and avoidance behavior, leading to increased healthcare utilization. These patients might benefit from help and support to evaluate the perception and management of their chest pain. The purpose of this study was to test the feasibility of a short guided Internet-delivered cognitive behavioural therapy (CBT) program and explore the effects on cardiac anxiety, fear of body sensations, depressive symptoms, and chest pain in patients with non-cardiac chest pain, compared with usual care.

**Methods:**

A pilot randomized controlled study was conducted. Fifteen patients with non-cardiac chest pain with cardiac anxiety or fear of body sensations, aged 22–76 years, were randomized to intervention (*n* = 7) or control (*n* = 8) groups. The four-session CBT program contained psychoeducation, physical activity, and relaxation. The control group received usual care. Data were collected before and after intervention.

**Results:**

Five of seven patients in the intervention group completed the program, which was perceived as user-friendly with comprehensible language, adequate and varied content, and manageable homework assignments. Being guided and supported, patients were empowered and motivated to be active and complete the program. Patients in both intervention and control groups improved with regard to cardiac anxiety, fear of body sensations, and depressive symptoms, but no significant differences were found between the groups.

**Conclusions:**

The Internet-delivered CBT program seems feasible for patients with non-cardiac chest pain, but needs to be evaluated in larger groups and with a longer follow-up period.

**Trial registration:**

Clinicaltrials.gov NCT02336880. Registered on 8 January 2015.

**Electronic supplementary material:**

The online version of this article (doi:10.1186/s13063-016-1491-1) contains supplementary material, which is available to authorized users.

## Background

More than 50 % of patients seeking hospital care because of chest pain are diagnosed as having “non-cardiac” symptoms [1, 2]. Many patients are discharged without knowing the cause of their chest pain [3, 4]. Patients with non-cardiac chest pain experience psychological distress [5, 6] and use healthcare resources to a great extent, leading to high healthcare costs [7–11]. Despite reassurance [12], many patients think they have an undetected cardiac disease and avoid activities that they believe might be harmful to the heart [13, 14]. Cardiac anxiety is common in patients with recurrent non-cardiac chest pain [6], leading to a vicious cycle, as it leads to maintenance of both anxiety and pain and secondary avoidance of physical activity [15, 16].

Targeting cardiac anxiety with psychological interventions might break the vicious circle and improve patient outcomes. Patients need to evaluate the way they perceive and handle their chest pain; this can be achieved using cognitive behavioural therapy (CBT) [17]. There is strong support for face-to-face CBT in the treatment of mild and moderately severe states of anxiety and depressive disorders [18–20] and non-cardiac chest pain [21, 22]. Previous face-to-face CBT studies [12, 23–26] have shown positive effects on chest pain frequency, activity avoidance, anxiety, and depression among patients with non-cardiac chest pain.

Face-to-face CBT can be effective when delivered by experts, but it is time-consuming [27, 28] and therefore not easy to provide to everyone. Internet-delivered CBT seems as a good alternative since it is cheaper, is not time dependent, and requires less therapist involvement [28], and could therefore be given to more patients. Therapist-guided Internet-delivered CBT does not differ from face-to-face treatment with regard to treatment effects [28–30], regardless of the background of the therapist guiding the Internet-delivered CBT [18, 31–33]. As concluded by Sharp et al. [34], Internet-delivered CBT might be a means to increase access to psychological treatment to patients with different chronic somatic conditions, although more research is needed to establish the feasibility and efficacy of Internet-delivered CBT for such populations. However, no Internet-delivered CBT programs have been tested in patients with non-cardiac chest pain. Our hypothesis was that a guided Internet-delivered CBT program targeting cardiac anxiety can help patients modify their beliefs about chest pain, change their cognitive and behavioural strategies, and give them tools to handle their chest pain. Furthermore, a four-week treatment would be preferred by the patients and easier to implement in the healthcare settings than a longer face-to-face treatment. The purpose of the study was to test the feasibility of a short guided Internet-delivered CBT program and explore the effects on cardiac anxiety, fear of body sensations, depressive symptoms, and chest pain in patients with non-cardiac chest pain, compared with usual care.

## Methods

### Study design

The study was designed as a pilot randomized controlled study and registered at Clinicaltrials.gov, no. NCT02336880. The study is reported in accordance with the CONSORT 2010 checklist of information to include when reporting a randomized trial (see Additional file 1). Participants were randomly assigned to either an intervention or a control group using a randomization table provided by a statistician not involved in the study.

### Participants

Eligible for the study were patients over the age of 18 who had sought medical care at least three times during the previous 6 months because of non-cardiac chest pain (International Classification of Diseases 10 codes: R07.2, R07.3, R07.4, and Z03.4), and who had cardiac anxiety (≥24 points on the Cardiac Anxiety Questionnaire), or fear of body sensations (≥28 on the Body Sensations Questionnaire). These cut-off scores were based on results from our earlier study [6]. Exclusion criteria were: no access to computer, tablet, or Internet, inability to perform physical activity, language difficulties, or severe depressive symptoms.

### Internet-delivered CBT program

**Fig. 1:**
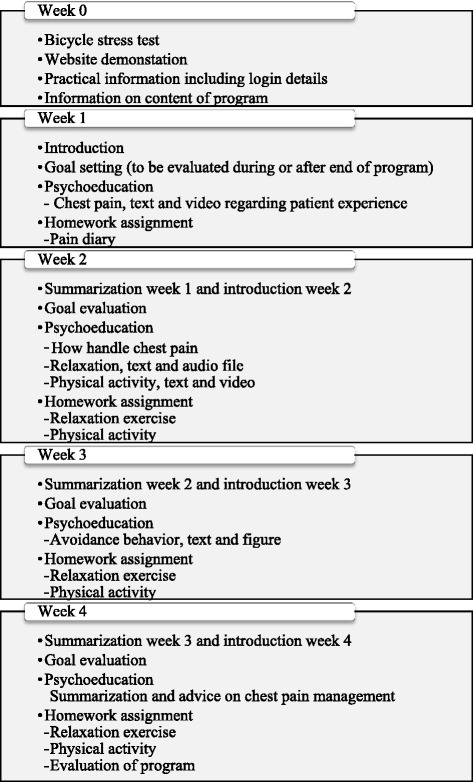
Content of CBT program

The CBT-based program contained four sessions, focusing on the behavioural aspects of CBT, intended to be completed in four weeks. The program was influenced by the intervention by Jonsbu et al. [14] and included exposure to physical activity, starting with a bicycle stress test, goal setting, psychoeducation, a breathing-based relaxation exercise, and a weekly homework assignment (see Fig. 1). The program was completed using a secure Internet platform, i.e., only those who received the website URL and login details had access to the program. For login, a two-factor authentication was applied using Google Authenticator. The Internet platform offered introductory information about the goals and the content of the program, how to handle the program, the names and photos of the study team, as well as contact details for the first author. Once logged in, patients could access one session each week. Every session started with a summary of the key knowledge from the previous session and the content of the following session. All sessions had one or more homework assignments that patients submitted at the end of each session. Before the start of the study, the program was reviewed individually by a general practitioner and two patients with long experience of non-cardiac chest pain.

#### Psychoeducation on chest pain

Psychoeducation has shown to be effective in the treatment of many conditions, including health anxiety and depressive symptoms [35, 36]. The patients were provided with information on chest pain, potential causes, chest pain management, avoidance behavior, and how this can maintain or exacerbate chest pain. For homework, patients were asked to complete a pain diary developed for this study during the first week to learn more about their chest pain and its impact on daily life. This included situations that they thought had caused the chest pain, emotions and reactions experienced, and thoughts that arose in connection with chest pain. They were also asked to reflect on and note in the diary what strategies they used to handle chest pain.

#### Exposure to physical activity

Exposure to tasks that the individual misinterprets as threatening is a central component to treat anxiety and relieve fear avoidance [16]. Exposure to physical activity has shown to be effective in the treatment of anxiety in patients with non-cardiac chest pain [14]. Therefore, the intervention group was exposed to physical activity, starting with a bicycle stress test, to reassure them that their hearts tolerated physical activity. The stress test was done as an ergometer-based ramp test with continuous monitoring of electrocardiogram and estimation of exertion according to the Borg Rating of Perceived Exertion scale, ranging from 6 (no exertion at all) to 20 (maximal exertion) [37]. All tests were driven to exhaustion and were performed by an experienced cardiologist. No symptoms, including significant cardiac ischemia or contraindications for continuing the test, were found.

The patients received psychoeducation about physical activity according to *Physical Activity in the Prevention and Treatment of Diseases* [38]. They were also provided with an eight-minute video, in which a physiotherapist gave instructions about physical activity. Patients were instructed to gradually increase the intensity to be able to perform moderate physical activity for at least 30 minutes per day, 5 days per week during the last 3 weeks of the program. For homework, they were asked to keep an activity diary, including the date, time, length, and type of activity, how the activity was perceived and what thoughts it conjured up, and the perceived exertion according to the Borg Rating of Perceived Exertion scale. By recording this information, patients could easily follow their own progress and increase their knowledge of the positive impact of physical activity, and thereby become motivated to continue being physically active.

#### Breathing-based relaxation exercise

Breathing technique has demonstrated a positive impact on the experience of anxiety, depressive symptoms, and the thinking pattern in relation to stress, thus leading to chest pain prevention [24, 39, 40]. Patients were provided with psychoeducation about relaxation, including an eight-minute long audio file of a breathing-based relaxation exercise called *The Breathing Anchor* [41]. The patients were instructed to perform the relaxation exercise using the audio file for at least 5 days per week during the last 3 weeks of the program. For homework, patients were asked to keep a relaxation diary including the date and time for relaxation, relaxation rate before and after the exercise on a scale ranging from 0 (not relaxed at all) to 10 (totally relaxed), and how they perceived the exercise. By recording their relaxation, patients could easily follow their own progress and become motivated to continue with the relaxation exercise.

#### Guiding and feedback

The patients were guided and supported through the program by the first author, who is a cardiac nurse with several years of teaching experience, and they received responses to their questions by email, text messages, and sometimes phone calls within 24 hours. Reminders and encouraging messages were also sent by email and text messages to motivate the patients to complete the intervention [28, 42]. Patients received individualized feedback on their goals and were also provided with weekly feedback on their homework assignments. Feedback was provided by email by the first author and concerned the positive progress patients made during the intervention, as well as encouragement to proceed with the program. We chose to give patients feedback, since it is a crucial part of Internet-delivered CBT [28].

### Control condition

The control group received usual care, meaning that they could seek healthcare within primary and secondary care settings whenever needed.

### Procedure

Study patients were recruited using a regional care database run by the County Council of Östergötland, Sweden. A list of names of patients fulfilling the inclusion criteria was given to the first author, who contacted the patients by phone and informed them of the study. Written study information, an informed consent form, and a pre-stamped envelope were sent to patients who were interested in participation. Patients who returned a signed written informed consent form were screened for cardiac anxiety or fear of body sensations before randomization.

Data were collected before randomization (i.e., baseline) and after the CBT program. All data were self-reported and collected using secure web-based questionnaires requiring both username and password for login. All questions were mandatory, resulting in no missing values.

### Data collection and measurement

#### Feasibility

Feasibility of the study concerned recruitment and randomization of study participants for sufficient sample size, testing the study protocol, data collection, and evaluation of selected outcomes. Feasibility of the intervention, however, concerned the user-friendliness of the Internet platform, the time and amount of guidance needed to manage the program, the accessibility of the content, and the patients’ perception of the program. Feasibility data were collected after the end of the program using a self-developed questionnaire containing 18 questions and statements regarding the aforementioned parts of feasibility and how this could be enhanced.

#### Cardiac anxiety

The Cardiac Anxiety Questionnaire was used to assess cardiac anxiety. This questionnaire comprises 18 items, each scoring between 0 and 4 points and consists of three subscales for fear, avoidance, and heart-focused attention. A sum and a mean total score can be calculated for the Cardiac Anxiety Questionnaire. For the subscales, the mean score of the items included in each subscale is preferable, since these differ in number. Higher scores indicate greater cardiac anxiety and, for the subscales, greater fear, avoidance and heart-focused attention. The Cardiac Anxiety Questionnaire has demonstrated good psychometric properties [43]. Cronbach’s *α* coefficients in this study were, at baseline, 0.83 for the total scale, and 0.83, 0.87, and 0.65 for the three subscales.

#### Fear of body sensations

Fear of body sensations was measured with the Body Sensations Questionnaire, comprising 17 items, each scoring between 1 and 5 points. Total score is computed as the mean value of all items, with higher scores indicating more fear of body sensations. The Body Sensations Questionnaire is reliable and valid [44]. Cronbach’s *α* coefficient was 0.92 in this study.

#### Depressive symptoms

Depressive symptoms were measured with the Patient Health Questionnaire-9. This is a nine-item questionnaire, with items scoring between 0 and 3 points. A score of 10 or higher indicates at least moderate depressive symptoms. The Patient Health Questionnaire-9 has demonstrated good psychometric properties [45]. In this study, Cronbach’s *α* coefficient was 0.85.

#### Chest pain frequency

Chest pain frequency was determined by asking the patients the following self-developed question: *“*During the last month, how often have you experienced non-cardiac chest pain?”

### Statistical analysis

IBM SPSS Statistics 22 was used in all statistical analysis. Non-parametric statistics were used, owing to the small sample and skewed variables. Categorical variables are described in numbers and percentages, and were analyzed with chi-square tests. Continuous variables are described in medians and ranges, and analyzed with the Mann–Whitney *U* test. Mean values and standard deviations were used to report the scores of the measurements. To determine the differences between groups, Mann–Whitney *U* test was used to analyze mean differences in cardiac anxiety, fear of body sensations, and depressive symptoms between baseline and the end of the program. All analyses were based on intention to treat. Differences were considered significant at *P* < 0.05.

## Results

### Participants

**Fig. 2:**
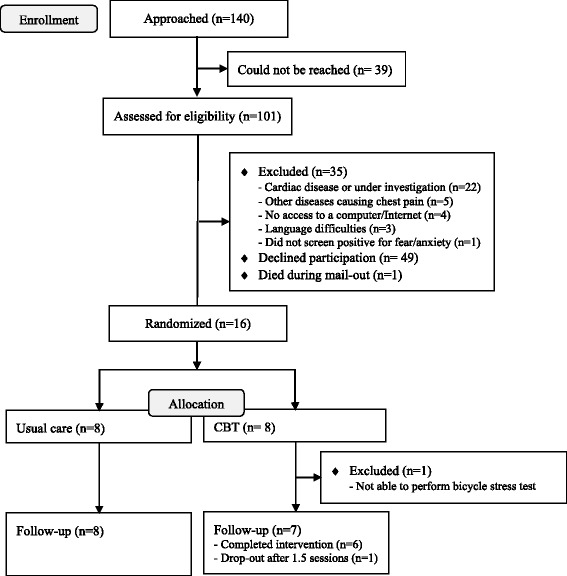
CONSORT flow chart of recruitment and follow-up process

A total of 101 patients were assessed for eligibility (see Fig. 2 for CONSORT flow chart). Fifteen patients fulfilled the inclusion and exclusion criteria and were randomized; seven in the intervention and eight in the control group. Demographic data and previous diseases or health complaints for these patients are presented in Tables 1 and 2.

**Table 1:** Demographic data in study patients at baseline, *N* = 15

	Intervention (*n* = 7)	Control (*n* = 8)	*P*
Age, years; median (range)	65 (22–76)	66 (53–75)	0.816
Sex *n* (%)			0.398
Male	5 (71)	4 (50)	
Female	2 (29)	4 (50)	
Birth country *n* (%)			0.268
Sweden	6 (86)	8 (100)	
Other Nordic countries	1 (14)		
Relationship status *n* (%)			0.935
Married or cohabiting	5 (71)	5 (63)	
Single	2 (29)	3 (38)	
Educational level *n* (%)			0.689
Compulsory school	1 (14)	1 (13)	
High school	4 (57)	3 (38)	
University	2 (29)	4 (50)	
Work status *n* (%)			0.438
Worker	3 (43)	3 (38)	
Retired	3 (43)	2 (25)	
Sick-leave or disability pension	1 (14)	2 (25)	
Part-time disability pension	0 (0)	1 (13)	
Smoking *n* (%)			0.186
None or previous smokers	6 (86)	6 (75)	
Smokers	1 (14)	2 25)	
Alcohol consumption *n* (%)			0.469
None	3 (43)	3 (38)	
1–7 glasses/week	3 (43)	4 (50)	
> 7 glasses/week	1 (14)	1 (13)	
Exercise *n* (%)			0.027
0 hours/week	1 (14)	5 (63)	
< 1 hour/week	1 (14)	2 (25)	
1–3 hours/week	3 (43)	1 (13)	
> 3 hours/week	2 (29)	0 (0)	
Experience of non-cardiac chest pain, months; median (range)	3 (1–360)	18 (3–612)	0.103

**Table 2:** Self-reported diseases or complaints in patients with non-cardiac chest pain, *N* = 15

	Number	
Diseases or complaints	Intervention group, *n* = 7	Control group, *n* = 8
Anxiety or panic disorder	4	4
Hypertension	4	6
Musculoskeletal pain	4	3
Asthma or bronchitis	4	1
Depression	3	1
Myocardial infarction	2	2
Reflux or heartburn	2	5

The intervention group consisted of five men and two women with a median age of 65 years (range 22–76). The control group was equal with regard to sex, and had a median age of 66 years (range 53–75). The intervention group only differed from the control group regarding number of exercise hours per week, as the intervention group exercised more hours (*P* = 0.027). Non-participants (*n* = 125) consisted of 71 men and 54 women and had a median age of 68 years (range 23–98). These did not differ significantly regarding sex and age, compared with study participants.

### Feasibility

Recruited patients were easily randomized into either the intervention or control group using a randomization table. Five out of the seven patients randomized to the intervention completed all sessions as planned. One participant completed only the parts of the program that he thought were needed. Despite several reminders and one week’s extended intervention time (5 weeks in total), the participant did not become more active. Another participant who had been very active during the start of the intervention dropped out after 1.5 weeks (sessions), owing to difficulties with the technology.

The study protocol was easy to follow and all patients were guided by the same person. Patients reported working between 30 minutes and 90 minutes per day on the program, with a median of 60 minutes. On average, about 22 minutes’ weekly therapist time was required to guide, support, and give feedback to each patient through the program. Two of the patients required about 35 minutes each, owing to inactivity, which resulted in repeated contacts.

The program was evaluated as user-friendly, with comprehensible language, adequate and varied content (i.e., text, videos, and audio file), and manageable homework assignments, but could benefit from more tailored exercises and assignments. Furthermore, there was a need for some technology support, i.e. how to handle the two-factor authentication, switching between the different parts of the program, and filling in the forms.

Almost all patients thought that the program had met their expectations and provided valuable knowledge and tools, and they thought it was worth the time and effort they invested in the program. Four of the patients reported that the program helped them reach their goals to a great extent. Getting reassured that their hearts tolerated physical activity made some patients less worried and anxious about their chest pain. Participation in the program, and particularly being guided and supported, motivated and empowered some patients to be active, despite cardiac anxiety. Some patients felt an obligation to complete the program because they were surveyed, which they did despite sometimes feeling tired and not in the mood. Anyway, patients were in general satisfied with the overall program and were willing to recommend it to others.

### Changes in cardiac anxiety, fear of body sensations, depressive symptoms, and chest pain frequency

**Fig. 3:**
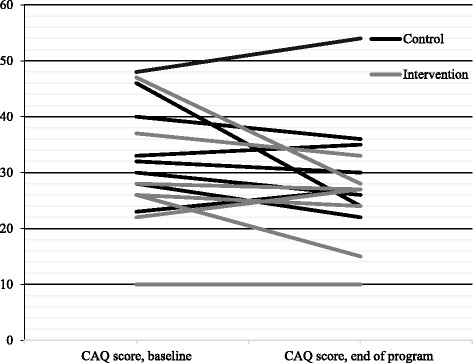
Cardiac anxiety (Cardiac Anxiety Questionnaire score) at baseline and at the end of the program, presented for each patient

**Fig. 4:**
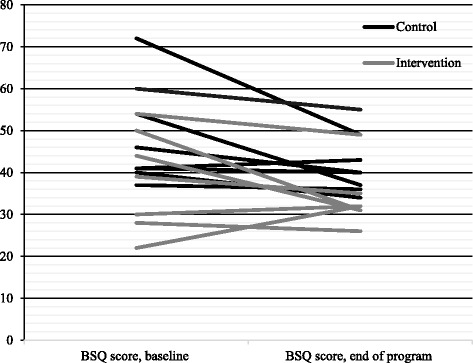
Fear of body sensations (Body Sensations Questionnaire score) at baseline and at the end of the program, presented for each patient

**Fig. 5:**
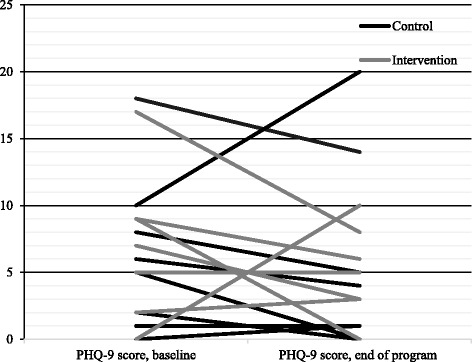
Depressive symptoms (Patient Health Questionnaire-9 score) at baseline and at the end of the program, presented for each patient

Cardiac anxiety, fear of body sensations, and depressive symptoms decreased in patients in both intervention and control groups (Table 3). There were no significant differences between the groups regarding mean differences between baseline and the end of the program. Figures 3, 4, and 5 demonstrate the changes per patient between baseline and end of the program. In the intervention group, five patients reported lower Cardiac Anxiety Questionnaire scores (i.e., less cardiac anxiety) at the end of the program, one reported higher scores, and one did not change. In the control group, five patients reported lower Cardiac Anxiety Questionnaire scores and three patients reported higher Cardiac Anxiety Questionnaire scores.

**Table 3:** Cardiac Anxiety Questionnaire, Body Sensations Questionnaire, and Patient Health Questionnaire-9 scores at baseline and at the end of the program, and comparison between intervention and control group regarding mean differences in these scores between baseline and at the end of the program

Variables	Mean ± standard deviation						
	Intervention (*n* = 7)			Control (*n* = 8)			
	Baseline	End of program	Mean differences	Baseline	End of program	Mean differences	*P* ^a^
Cardiac Anxiety Questionnaire							
Total score	28.0 ± 11.6	23.4 ± 8.1	−4.6 ± 8.0	35.0 ± 8.8	31.8 ± 10.3	−3.3 ± 8.7	0.86
Mean score	1.6 ± 0.6	1.3 ± 0.4	−0.3 ± 0.4	1.9 ± 0.5	1.8 ± 0.6	−0.2 ± 0.5	0.86
Fear	2.0 ± 0.9	1.7 ± 0.6	−0.2 ± 0.8	2.3 ± 0.8	2.0 ± 0.9	−0.3 ± 0.5	0.64
Avoidance	1.2 ± 0.8	0.9 ± 0.7	−0.3 ± 0.6	1.7 ± 1.0	1.7 ± 1.2	−0.1 ± 0.6	0.32
Heart-focused attention	1.2 ± 0.8	1.0 ± 0.5	−0.2 ± 0.6	1.6 ± 0.5	1.4 ± 0.3	−0.2 ± 0.7	0.86
Body Sensations Questionnaire							
Total score	38.1 ± 12.0	33.7 ± 7.3	−4.4 ± 9.5	48.9 ± 12.2	41.8 ± 7.1	−7.1 ± 8.6	0.56
Mean score	2.2 ± 0.7	2.0 ± 0.4	−0.3 ± 0.6	2.9 ± 0.7	2.5 ± 0.4	−0.4 ± 0.5	0.56
Patient Health Questionnaire-9							
Total score	7.0 ± 5.6	5.0 ± 3.4	−2.0 ± 6.6	6.3 ± 5.9	5.6 ± 7.4	−0.6 ± 4.7	0.60

^a^Differences between mean differences in the intervention and control groups

Five patients in the intervention group reported lower Body Sensations Questionnaire scores (i.e., less fear of body sensations) at the end of the program and two reported higher scores. In the control group, seven patients reported lower scores and one reported higher scores. Regarding depressive symptoms, four patients in the intervention group reported lower Patient Health Questionnaire-9 scores at the end of the program, two reported higher scores, and one did not change. In the control group, five patients reported lower scores, two reported higher scores, and one did not change. With regard to minimal significant change of five points on the Patient Health Questionnaire-9, two patient scores decreased by nine points in the intervention group, whereas in the control group, one person’s score decreased by five points.

At baseline, three patients in the intervention group reported daily chest pain and two reported chest pain at least twice per week. The rest had chest pain sporadically. At the end of the program, one patient reported daily chest pain and three reported chest pain at least twice per week. The rest had chest pain sporadically. Compared with baseline, two patients had less chest pain and five did not change at the end of the program. In the control group, four patients reported daily chest pain and one reported chest pain at least twice per week, both at baseline and at the end of the program. The other patients had chest pain sporadically. At the end of the program, one patient had less chest pain, one had more chest pain, and six did not change, compared with baseline.

## Discussion

This is the first study assessing the feasibility and effects of a short guided Internet-delivered CBT program in patients with non-cardiac chest pain. Patients perceived the program to be feasible and user-friendly, with comprehensible language, adequate and varied content, and manageable homework assignments, although some patients needed support with the technology. The program showed the potential to help patients with non-cardiac chest pain improve regarding cardiac anxiety, fear of body sensations, depressive symptoms, and chest pain frequency, but this was not significantly different from the control group. The results of this study can contribute to the design of future interventions to test the effectiveness of Internet-delivered CBT programs in these patients.

Regarding treatment and technology, our patients’ levels of knowledge, needs, and preferences varied. One participant only completed parts of the program, despite repeated phone and email contacts. Another participant had technology-related difficulties and dropped out of the program. This problem has been pointed out as one of the disadvantages with Internet-delivered treatments [29]. More support might be needed for those who have technology-related difficulties and for those participants, alternative treatments, such as face-to-face meetings should be considered. Since we only evaluated Internet-delivered CBT, this was not an alternative in our study. Non-adherence and non-completion of both Internet-delivered and face-to-face interventions are very common and have similar underlying reasons as in our study [29, 46–48].

On average, only 22 minutes’ therapist time per week was needed to guide, support, and give feedback to an ordinary patient through the program. Other Internet-delivered CBT studies have reported feedback times of between 9 and 20 minutes [46, 49, 50], but it is unclear whether this only included feedback. With regard to time, other aspects to consider are that our intervention was new and the patients were guided by a therapist not trained in CBT, which could have had an impact on the time for guiding and feedback. Along with more experience, improved performance can be achieved over time [51]. Being guided by the same person could ensure that the same intervention was delivered to all patients and that patients were adhering to the study protocol and understanding what was expected from them.

With regard to the effects of the intervention, some patients reported being reassured by the bicycle stress test that their hearts tolerated physical activity, which made them less worried and anxious about chest pain. This is an important finding, since modification of thoughts and behaviors can lead to decreased avoidance of physical activity, less anxiety, and also chest pain in these patients [16, 52]. Since many of the patients undergo some kind of stress test to rule out cardiac disease, physicians could use the test to reassure patients with non-cardiac chest pain that they can be physically active despite chest pain, rather than just telling them that they do not have cardiac disease. This might decrease cardiac anxiety and avoidance of physical activity.

Since Internet-delivered CBT has demonstrated a potential to decrease anxiety, depression, and pain in various patient groups [27, 31, 53, 54], we assumed that this could be applied also in our setting. Most of the patients in our study, both in the intervention and control groups, improved and reported decreased scores in cardiac anxiety, fear of body sensations, and depressive symptoms, and only a few of them reported increased scores. The small changes in pain found in the intervention group might be due to more knowledge about chest pain and reassurance regarding their hearts tolerating physical activity, and also to relaxation capability. By reducing pain catastrophizing, fear avoidance, and cardiac anxiety, pain reduction can be achieved [55, 56]. The patients in the intervention group reported a slightly greater decrease on the cardiac anxiety and depressive symptom scales than did the patients in the control group, but this was not statistically significant. This is possibly because the samples were small. It could also result from the early follow-up or perhaps the intervention were too short. Normally, face-to-face CBT ranges between 4 and 16 weekly sessions, although most are about 8 to 12 sessions long [17, 48]. Therefore, it might be that four weeks are too short to enable patients to modify their thoughts and change their behaviors, although one study was successful despite having a program that was shorter by one week [14]. Moreover, the effects of the CBT program were only evaluated at the end of the program; thus, the long-term effects of the CBT program are unknown. Hence, the length of the program, and also when to follow-up the intervention need to be studied further.

Our results are comparable with results from a previous study by Sanders et al. [57], using a brief CBT intervention with a 1 hour information and discussion session, using a booklet on chest pain and how to cope with it, a cassette tape with breathing and relaxation exercises, and a telephone follow-up every two weeks. At the 3-month follow-up, they found no differences between the treatment and control groups. This was partly explained by many drop-outs, leading to a small sample. In our study, not all patients improved, and some patients in both the intervention and control groups reported a slight increase in cardiac anxiety, fear of body sensations, depressive symptoms, and chest pain frequency between baseline and the end of the program. Since this was the case for both groups, the deterioration could not be related to the intervention. Similar conclusions were drawn by Zou et al. [30].

Recruitment to this study was a challenge. One of the reasons might be that patients may have considered their pain to have cardiac or other physical causes and therefore declined participation, since they perceived our intervention as too psychological and not really addressing their perceived physical cause of their chest pain, as previously reported by Sanders et al. [57]. Additionally, patients might have estimated the program to be too burdensome and declined participation. Similar barriers were identified by Tyrer et al. [58]. Approaching patients with whom the researchers did not have a care relation could have affected patients’ willingness to participate. We assume that patients might feel more motivated to participate in a research study if informed by a person with whom they have a care relation. We therefore suggest that future studies should prospectively recruit patients within clinics at discharge to overcome these barriers. This requires involvement of well-informed and motivated nurses and physicians who can reinforce the pointlessness of continued hospital visits and encourage patients to join such interventions. The many criteria for inclusion also led to a decreased number of eligible patients. One way to gain access to a greater number of eligible patients is to conduct multicenter studies with long recruitment periods.

### Limitations

The study is a pilot and based on a small sample with differences in length of chest pain experience between the intervention and control groups, although these were not significant. However, the results should be interpreted with caution. There might also have been a selection bias, since those interested in participation were more healthy and receptive to this kind of psychological treatment. Some of the barriers for participation were that some patients did not have access to the Internet or did not feel comfortable handling computers. Only pain frequency was measured. We suggest that pain intensity should be included in future studies.

## Conclusions

A short guided Internet-delivered CBT intervention was perceived to be feasible. The CBT intervention was found to decrease cardiac anxiety, fear of body sensations, depressive symptoms, and chest pain frequency, but no significant differences were found compared with the control group. Larger studies with longer follow-up are needed to further evaluate both the short- and long-term effects of Internet-delivered CBT in patients with non-cardiac chest pain.

## Abbreviations

CBT, cognitive behavioural therapy; CONSORT, Consolidated Standards of Reporting Trials

## Additional file

Additional file 1: CONSORT 2010 checklist of information to include when reporting a randomized trial. (DOC 217 kb)
